# Clinico-Pathological Discrepancies in the Diagnosis of Causes of Maternal Death in Sub-Saharan Africa: Retrospective Analysis

**DOI:** 10.1371/journal.pmed.1000036

**Published:** 2009-02-24

**Authors:** Jaume Ordi, Mamudo R Ismail, Carla Carrilho, Cleofé Romagosa, Nafissa Osman, Fernanda Machungo, Josep A Bombí, Juan Balasch, Pedro L Alonso, Clara Menéndez

**Affiliations:** 1 Department of Pathology Hospital Clinic, Universitat de Barcelona, Institut d'investigacions biomèdiques August Pi I Sunyer (IDIBAPS), Barcelona, Spain; 2 Barcelona Centre for International Health Research (CRESIB), Hospital Clinic/Universitat de Barcelona, IDIBAPS, Barcelona, Spain; 3 Department of Pathology, Maputo Central Hospital, Universidad Eduardo Mondlane, Maputo, Mozambique; 4 Manhiça Health Research Center (CISM), Manhiça, Mozambique; 5 Department of Obstetrics and Gynecology, Hospital Central de Maputo, Universidad Eduardo Mondlane, Maputo, Mozambique; 6 Department of Obstetrics and Gynecology, Hospital Clinic, University of Barcelona, Barcelona, Spain; National Institute of Child Health and Human Development, United States of America

## Abstract

**Background:**

Maternal mortality is a major public-health problem in developing countries. Extreme differences in maternal mortality rates between developed and developing countries indicate that most of these deaths are preventable. Most information on the causes of maternal death in these areas is based on clinical records and verbal autopsies. Clinical diagnostic errors may play a significant role in this problem and might also have major implications for the evaluation of current estimations of causes of maternal death.

**Methods and Findings:**

A retrospective analysis of clinico-pathologic correlation was carried out, using necropsy as the gold standard for diagnosis. All maternal autopsies (*n =* 139) during the period from October 2002 to December 2004 at the Maputo Central Hospital, Mozambique were included and major diagnostic discrepancies were analyzed (i.e., those involving the cause of death). Major diagnostic errors were detected in 56 (40.3%) maternal deaths. A high rate of false negative diagnoses was observed for infectious diseases, which showed sensitivities under 50%: HIV/AIDS-related conditions (33.3%), pyogenic bronchopneumonia (35.3%), pyogenic meningitis (40.0%), and puerperal septicemia (50.0%). Eclampsia, was the main source of false positive diagnoses, showing a low predictive positive value (42.9%).

**Conclusions:**

Clinico-pathological discrepancies may have a significant impact on maternal mortality in sub-Saharan Africa and question the validity of reports based on clinical data or verbal autopsies. Increasing clinical awareness of the impact of obstetric and nonobstetric infections with their inclusion in the differential diagnosis, together with a thorough evaluation of cases clinically thought to be eclampsia, could have a significant impact on the reduction of maternal mortality.

## Abstract 2

Jaume Ordi and colleagues examine the discrepancies between clinical diagnoses of causes of maternal deaths and pathological findings by necropsy in Mozambique.

## Editors' Summary

**Background.:**

Every year, about half a million women die during pregnancy or childbirth or soon after delivery—so-called “maternal deaths.” Although nearly all these maternal deaths occur in developing countries, the situation is particularly bad in sub-Saharan Africa where more than a quarter of a million maternal deaths occur annually. The number of maternal deaths per 100,000 live births in this region is nearly 1,000, whereas in developed regions it is only nine deaths. A 15-year-old girl living in sub-Saharan Africa has a lifetime risk of dying during pregnancy or childbirth of 1 in 22, but a girl living in the developed regions of the world has a lifetime risk of only 1 in 7,300. Maternal deaths can be caused by obstetric (childbirth-related) complications such as puerperal septicemia (an infection of the blood contracted during delivery) and eclampsia (seizures associated with high blood pressure during pregnancy), and by nonobstetric conditions such as HIV/AIDS-related infections and other infections.

**Why Was This Study Done?:**

In 2000, the United Nations made reduction of the global burden of maternal mortality one of its Millennium Development Goals (a set of targets designed to eradicate poverty by 2015), but little progress has been made toward achieving this goal. One possible explanation for this failure might be that limited access to diagnostic tests in developing countries results in more clinical diagnostic errors than in developed countries and that, consequently, mothers in developing countries don't always get the right treatment when they become ill. Unfortunately, it is difficult to test this hypothesis, because there is very little accurate information on the causes of maternal death in many developing countries. What information there is comes mainly from clinical records and verbal autopsies (asking relatives about the mother's death) rather than from examination of the body after death (a medical autopsy), the only sure way to ascertain the cause of death. In this study, the researchers retrospectively analyze discrepancies between the clinical diagnoses and autopsy diagnoses of 139 mothers who died at the Maputo Central Hospital, Mozambique, a large hospital providing specialized care for women with high-risk pregnancies.

**What Did the Researchers Do and Find?:**

All the organs from the mothers were visually examined by a pathologist and samples of any abnormal tissues and of the internal organs were examined microscopically. Two pathologists independently established the cause of each death by considering both the clinical diagnosis and the autopsy results (the “autopsy diagnosis”). The discrepancies between the clinical and autopsy (“gold standard”) diagnoses were then analyzed. Major diagnostic errors (errors involving the cause of death) occurred in nearly half of the maternal deaths; the clinical and autopsy diagnoses completely agreed in only a third of cases. 80% of the major diagnostic errors were “class I errors.” That is, errors where a correct diagnosis would have changed patient management and prolonged survival or provided a cure. For example, 12 women were given an incorrect diagnosis of eclampsia when they had other conditions that could have been successfully treated if correctly diagnosed. Furthermore, many infections detected in the autopsies were missed in the clinical diagnoses (false-negative diagnoses), some of which could have been treated.

**What Do These Findings Mean?:**

These findings show that clinical and autopsy diagnoses of the causes of maternal death frequently disagree in this hospital. Further studies are needed to see whether similar levels of disagreement exist in other hospitals in sub-Saharan Africa. The discrepancy reported here might, for example, be an overestimate of the general situation, because the high-risk pregnancies referred to this hospital might involve more hard-to-diagnose problems than the routine pregnancies referred to other hospitals. Nevertheless, these findings suggest that misdiagnosed conditions may affect maternal mortality rates in sub-Saharan Africa and that an increased use of autopsy in the region could reduce maternal mortality by providing more accurate information about why mothers die. In particular, these findings suggest that a more thorough evaluation of cases thought to be eclampsia and a better awareness of the involvement of infectious diseases in maternal deaths might reduce diagnostic errors and consequently reduce the incidence of maternal deaths.

**Additional Information.:**

Please access these Web sites via the online version of this summary at http://dx.doi.org/10.1371/journal.pmed.1000036.

- UNICEF (the United Nations Children's Fund) provides information on maternal mortality and the WHO/UNICEF/UNFPA/The World Bank estimates of maternal mortality for 2005 by country
- The WHO/UNICEF/UNFPA/The World Bank estimates of maternal mortality in 2005 also provides full information about global maternal mortality
- The UK Department for International Development provides information about Millenium Development Goal 5: the improvement of maternal health
- The Partnership for Maternal, Newborn and Child Health provides information on maternal deaths (in several languages), including information on the situation in Mozambique

## Introduction

There is a general consensus that maternal mortality is a major health problem worldwide as well as a fundamental public health indicator [1]. The problem of maternal mortality is concentrated in low-income countries, particularly in sub-Saharan Africa, where one in 16 women die of pregnancy-related complications [2,3]. Reduction of this intolerable burden is one of the Millennium Development Goals set up by the United Nations in 2000 [2].

The main source of information on the causes of maternal deaths in developing countries, clinical records and verbal autopsies (VA) [4–13], have serious limitations due to the frequent discrepancies between the clinically presumed and the actual cause of death [14]. It has recently been claimed that there is an urgent need for studies focused on providing an accurate knowledge of the causes of maternal death in developing countries [15]. Autopsy studies may thus improve the knowledge of the causes of maternal death by increasing the accuracy of cause-of-death reports [16].

Postmortem examination is also an essential tool to improve overall clinical diagnostic performance, since clinicians can only diagnose diseases for which they have been looking. Thus, the analysis of false positive and false negative diagnoses is essential to evaluate and improve the diagnostic process [17,18]. Unfortunately, very little information exists on discrepancies between clinical and necropsy data in maternal mortality causes in developing countries [19]. As a consequence, very little data is available on the impact of medical errors on maternal mortality.

In order to evaluate clinical practice and diagnostic performance, which could help to reduce the problem of maternal deaths in developing countries, we conducted a retrospective study on the discrepancies between clinical and postmortem diagnosis in maternal deaths at the Maputo Central Hospital (MCH) in Mozambique.

## Material and Methods

### Study Area and Design

The characteristics of the study area have been previously described [20]. The study was conducted at the Maputo Central Hospital, a government-funded tertiary health facility that serves as the referral center for other hospitals in Southern Mozambique. All women fulfilling the standard definition of the World Health Organization (WHO) for a pregnancy-related death (i.e., death during pregnancy, delivery, or within 42 d after completion of a pregnancy, irrespective of the cause of death) between October 2002 and December 2004, and for whom the family had given verbal informed consent to perform the autopsy, were included in the study. The study protocol was approved by the National Mozambican Ethics Committee and the Hospital Clinic of Barcelona Ethics Review Committee.

A complete dissection with macroscopic evaluation of each organ was performed by a pathologist using a standardized macroscopic protocol. Samples of all grossly identified lesions and of all viscera were collected in each case for histological study. A blood sample (100 μl) was obtained from the inferior vena cava and stored on filter paper.

Clinical diagnoses were obtained from those listed by the clinician on the clinical process, after a complete revision of the case notes. The final diagnoses were established by two pathologists after reviewing the clinical process, the macroscopic protocols, and the histological slides. Major diagnoses were those involving the principal underlying cause of death [21]. Minor diagnoses were: antecedent disorders, related diagnoses, contributing causes, or other important disorders [21]. Clinical and necropsy diagnoses were grouped into different categories according to the International Classification of Disease, tenth revision (ICD-10). HIV/AIDS-related diseases included all cases with opportunistic infections or other conditions included in the CDC revised criteria [22]. In this category the diagnosis of the opportunistic infection causing death, and not only the diagnosis of HIV/AIDS, were considered in the evaluation of discrepancies. The diagnostic criteria for severe malaria have been described elsewhere [20].

### Assessment of Discrepancies between Clinical and Necropsy Diagnoses

Diagnostic discrepancies were classified following the classification of Goldman et al. [23], modified by Battle et al. [21], and as nonclassifiable cases [24]. Major discrepancies were those involving major diagnoses and were classified as class I or class II discrepancies. In class I, the knowledge of the diagnosis before death, would have led to changes in the management that could have prolonged the survival or cured the patient (e.g., pyogenic meningitis treated as eclampsia), while in class II the survival would have not been modified (e.g., fulminant hepatitis treated as septicemia or terminal AIDS with multiple opportunistic infections treated as a bacterial infection). Minor discrepancies were those involving minor diagnoses and were classified as class III (diseases with symptoms that should have been treated or would have eventually affected the prognosis, e.g., mild aspirative pneumonia in a patient with eclampsia) and class IV (nondiagnosed diseases with possible epidemiological or genetic importance, e.g., schistosomal infections). Correctly diagnosed patients were classified as class V. Class VI were nonclassifiable cases (necropsy unsatisfactory or with no clear diagnosis).

A single class of major discrepancy (I and II) was assigned to each maternal death. The discrepancies were independently evaluated by two investigators (CR, JO). When minor disagreements were detected (class I versus class II, or class III versus class IV), a consensus meeting was held involving a third investigator (JAB). If a major disagreement was detected (e.g., class I versus class III), a senior pathologist uninvolved in the study was consulted.

### Laboratory Methods

Tissue specimens were fixed in 10% buffered formalin for 2–15 d and embedded into paraffin wax using standard procedures. For each sampled tissue 4-μm sections were stained with haematoxylin and eosin (HE). Ancillary histochemical and immunohistochemical stains were performed to confirm or exclude specific lesions suspected on the HE stains.

As previously reported [20], HIV provirus was determined using the standard Amplicor HIV-1 kit (Roche) on blood collected onto filter paper. In 46 women, HIV status was assessed prior to death using the rapid test Determine HIV (Abbot Laboratories), and positive results were confirmed with Unigold HIV (Trinity Biotech). Malaria parasitaemia was assessed prior to death on thick and thin air-dried blood films, stained with Giemsa. In the autopsy material a histologic evaluation of malarial pigment was done with light microscopy under polarized light [25,26].

### Definitions and Statistical Methods

Sensitivity, specificity, positive predictive value (PPV), negative predictive value (NPV), and accuracy were calculated for the most frequent clinical diagnoses, considering the pathological diagnosis as the gold standard. Sensitivity was calculated as the proportion of true positives divided by the sum of true positives and false negatives. Specificity resulted from the proportion of true negative divided by the sum of true negatives and false positives. PPV was calculated as the number of true positive cases divided by the sum of true and false positives and NPV as the number of true negatives divided by the sum of true and false negatives. Accuracy was calculated as the sum of true positive and true negative diagnoses in each diagnostic category divided by all maternal deaths. False-negative diagnoses were defined as class I and II discrepancies for which the necropsy diagnosis was in the assessed diagnostic category but the clinical diagnosis was in another. False-positive diagnoses were cases with class I and II discrepancies, in which the clinical diagnosis was in the diagnostic category but not the necropsy diagnosis. Data were analyzed with the program STATA (Version 8.0, StataCorp). Differences between groups were analyzed with the χ^2^ statistical analysis.

## Results

During the study period, there were 179 maternal deaths. In 139 women (77.6%) a complete autopsy with adequate histological sampling as well as clinical information was available.

A major diagnostic discrepancy was detected in 56 (40.3%) maternal deaths; 47 (83.9%) of them were classified as class I, and nine (16.1%) as class II. A minor diagnostic discrepancy (class III or IV) was identified in 30 maternal deaths (21.6%). An additional minor discrepancy was identified in 24 maternal deaths with a major error. Thus, an overall number of 54 minor discrepancies (38.8%) were identified. In 45 maternal deaths (32.4%), there was complete agreement between the clinical and the autopsy diagnoses (class V). In eight maternal deaths (5.7%), no diagnosis was reached in the autopsy and the deaths were thus considered as not suitable for evaluation (class VI). Macroscopic examination alone detected 24/56 (42.9%) clinical major errors, while the remaining 32 errors were detected only in the histological study. Table 1 shows the causes of death detected in the autopsies, the number of clinically suspected diagnoses for each final autopsy diagnosis, and the number and percentage of major diagnostic errors.

**Table 1:**
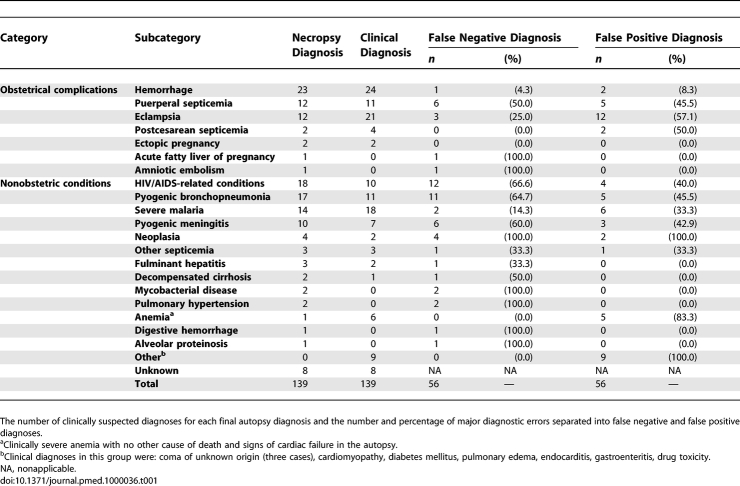
Causes of Death Detected in the Autopsies

Both evaluations of diagnostic discrepancy were coincident in 117 cases. A discordant evaluation was observed in 19 cases (13.7%), thus requiring a consensus. Sixteen of these disagreements were qualified as minor (eight class I versus class II; eight class III versus class IV). Three cases were qualified as major disagreements and required consultation with a senior pathologist.

**Figure 1:**
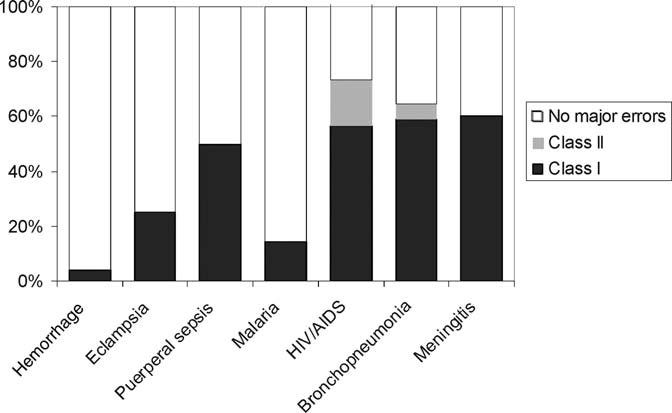
Prevalence of the Major Diagnostic Errors by Pathology at Autopsy

Seven diagnoses (obstetric hemorrhage, eclampsia, puerperal sepsis, HIV/AIDS-related conditions, pyogenic bronchopneumonia, malaria, and pyogenic meningitis), each responsible for ten or more maternal deaths, were further analyzed. Figure 1 shows the percentage of major errors (class I and II) in each of these diagnostic categories, and Table 2 the sensitivity, specificity, PPV, NPV, and accuracy of the clinical diagnosis for these seven categories.

**Table 2:**
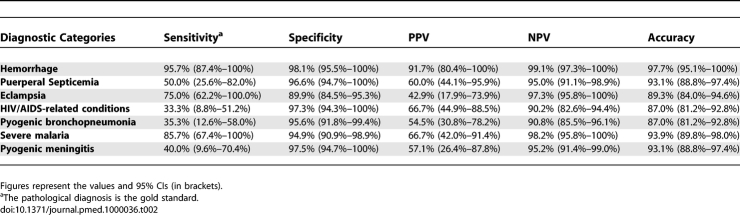
Sensitivity, Specificity, PPV, NPV, and Accuracy of the Clinical Diagnosis for All Frequent Diagnostic Categories

Disseminated tuberculosis was diagnosed in 12 women, of whom ten were HIV positive (and thus, included as HIV/AIDS-related condition). The diagnosis of tuberculosis had an overall sensitivity of 25.0% (95% confidence interval [CI]: 0.5%–49.5%), a specificity of 98.3% (95% CI: 96.0%–100%), a PPV of 60.0% (95% CI: 32.3%–87.7%), an NPV of 92.9% (95% CI: 88.3%–97.5%), and an accuracy of 91.6% (95% CI: 86.8%–96.4%).

Table 3 shows the reported clinical diagnoses in maternal deaths of obstetric hemorrhage, puerperal septicemia, eclampsia, pyogenic bronchopneumonia, severe malaria, and pyogenic meningitis with false negative diagnoses in the autopsy. The HIV positive status had been confirmed before death in eight out of 18 (44.4%) women with HIV/AIDS-related diseases, but only in six of them (33.3%) had the opportunistic infection directly causing the death been correctly diagnosed. Table 4 shows the false negative clinical diagnoses in maternal deaths with final diagnosis of HIV/AIDS-related diseases.

**Table 3:**
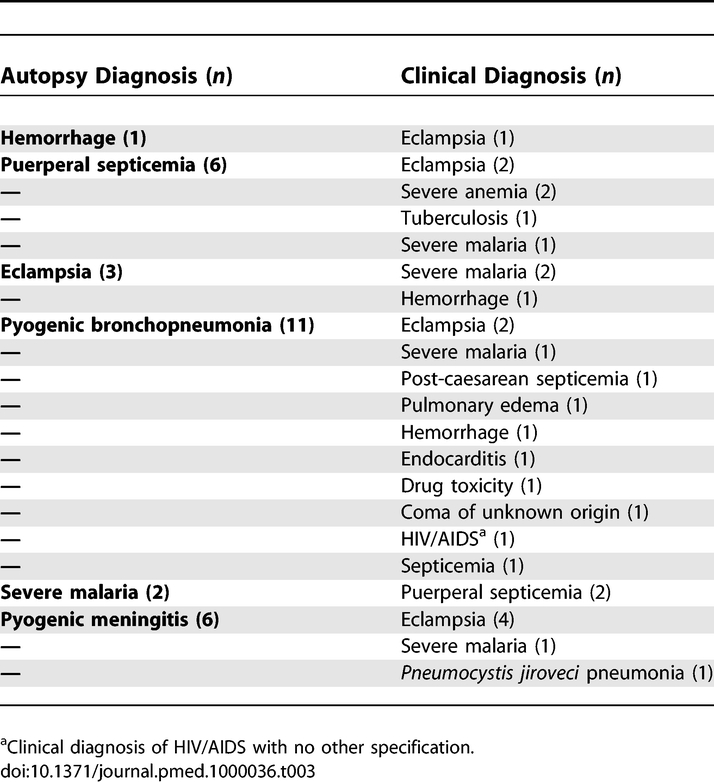
Clinical Diagnoses in Cases with False Negative Major Errors in Patients with Obstetric Hemorrhage, Puerperal Septicemia, Eclampsia, Pyogenic Bronchopneumonia, Severe Malaria, and Pyogenic Meningitis

**Table 4:**
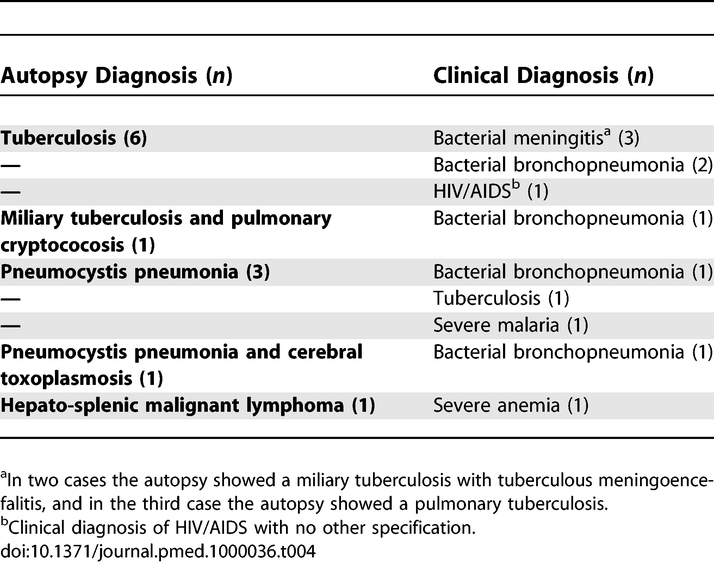
Clinical Diagnoses in Cases with False Negative Major Errors in Patients with HIV/AIDS Conditions as Cause of Death

False positive diagnoses were particularly frequent for eclampsia with 12 maternal deaths (Table 1). The autopsy diagnoses in maternal deaths with false positive clinical diagnosis of eclampsia were: pyogenic meningitis (four maternal deaths), meningioma (two maternal deaths), puerperal sepsis (two maternal deaths), pyogenic bronchopneumonia (two maternal deaths), tuberculosis (one maternal death), and postpartum hemorrhage (one maternal death). No pathological lesions related to eclampsia were detected in any of these women.

## Discussion

This is, to our knowledge, the first study focused on the evaluation of diagnostic discrepancies in maternal mortality in sub-Saharan Africa and even in developing countries, based on complete autopsies and including a histological study. This study has shown a high frequency of major clinico-pathological discrepancies (40.3%) in maternal deaths in a tertiary-referral hospital in sub-Saharan Africa. In most cases a change in clinical management could have significantly modified the prognosis. The proportion of discrepancies observed in this study is higher than that currently reported in studies based on nonselected hospital patients [17,27]. Remarkably, this study, unlike most previous studies, was based only on maternal deaths. These are extremely infrequent in developed countries [2,28], and tend to show lower discrepancy rates between clinical and autopsy diagnoses [16,21].

A sole description of the correlation between clinical and autopsy diagnosis was done retrospectively in Nigeria including data from 1989 to 1998 [19]. The prevalence of major diagnostic errors in that study was 10.4%, a figure much lower than that reported here. This fact may be explained mainly by methodological differences, since neither histological study nor HIV testing were used in the Nigerian study. Interestingly, in our study only 17.3% clinical major discrepancies were detected in the macroscopic study, whereas 57.1% of the discrepancies were found only in the histological study, as it has been shown in other studies [29,30].

The evaluation of sensitivity, specificity, PPVs, and NPVs may provide some insight into the possible factors behind major diagnostic errors. A high rate of false negative diagnoses was observed for infectious diseases, both obstetrical (puerperal septicemia) and nonobstetric. Other studies have shown that infectious diseases tend to show higher rates of diagnostic errors [21,31]. Interestingly, three frequent nonobstetric infectious categories (HIV/AIDS-related conditions, pyogenic bronchopneumonia, and pyogenic meningitis, as well as tuberculosis) had sensitivities below 40%. This number is very relevant since it indicates that significant reductions in maternal mortality could be reached by decreasing the false negative clinical diagnoses of some frequent infectious diseases, through improvements in their diagnosis. Although false negative diagnoses may occur either by omission, or lack of sensitivity of available diagnostic tests or inadequate synthesis in the diagnostic process [14,32,33], underestimation of prevalence plays an important role in this type of error [14,32,33]. It has been shown that increasing clinical awareness and the correct estimation of the prevalence of infectious diseases may lead to a reduction in the number of diagnostic errors [14,34]. Limited access to necessary diagnostic tests and particularly to microbiological cultures in developing countries, especially in sub-Saharan Africa, represents a major handicap for the diagnosis of many infectious diseases and significantly slows down the provision of drug therapy [35,36].

Eclampsia was the main source of false positive diagnoses (*n =* 12, 57.1%). It cannot be completely excluded that preeclampsia–eclampsia was a true diagnosis in some of these patients, and lack of an appropriate management of the condition a contributor to maternal death. However, a different cause of death was found in all these women. Moreover, no pathological lesions related to eclampsia were detected in any of them. False-positive diagnoses may occur due to premature closure of the diagnostic process and low specificity of diagnostic tests, but overestimation of prevalence also plays a major role in this type of error [14,32,33]. Generally, eclampsia is more likely to be diagnosed too frequently rather than overlooked. On the other hand, epilepsy, infections or space-occupying lesions of the central nervous system, cerebrovascular accidents, hypertensive diseases, metabolic disorders, and thrombotic thrombocytopenic purpura may all simulate eclampsia, but they are less frequent [37–39]. On the basis of clinical reports and VAs [4–12], eclampsia is widely accepted as one of the major causes of maternal death in sub-Saharan Africa [4,8,12]. The current study suggests that eclampsia remains a significant cause of maternal death in that setting, but its prevalence is probably overestimated.

The main limitation of our study is that it was conducted in a large university hospital located in the capital of Mozambique, which could limit the extrapolation of these findings to other hospitals and health facilities. It has been shown that the frequency of discrepancies between clinical and autopsy diagnoses is lower in larger hospitals [21], and this might suggest that the number of diagnostic errors in other health facilities would be underestimated. On the other hand, complicated or high risk pregnancies are often referred to these larger hospitals resulting in a high number of cases of increased diagnostic difficulty.

Four necessary conditions have been proposed for necropsy to be a valid monitor of clinical diagnosis performance: a high necropsy rate, specified and stable conditions of autopsy procedure (extent of organ assessment and sampling, availability of clinical information), calculation of sensitivity and specificity rather than accuracy, and estimate of the errors in postmortem diagnosis [40]. Our study met three of these conditions, but did not assess the error of autopsy diagnosis itself. The classification into the discrepancy classes was not clear-cut in all cases. A disagreement rate of 13.7% in class assignment was detected in our study, a figure that is similar to the disagreement rate reported in other studies [14]. This result could be seen as a limitation of the discrepancy classification.

The significant reduction of diagnostic errors reported in developed countries during the second half of the 20th century has been mainly attributed to the improvement of clinical skills and to the impact of new diagnostic procedures [14]. Necropsy has had in this regard the dual role of a method to detect diagnostic errors and a source of knowledge to be applied to future cases. This dual role has not only influenced learning but has also added relevant data on local epidemiology of diseases. The scientific, public health, and educational benefits of the autopsy remain difficult to evaluate, but are generally acknowledged [41]. The almost complete absence of studies based on necropsy data and focused on diagnostic error is a severe handicap for medical practice in sub-Saharan Africa. Thus, autopsy could help to reduce maternal mortality by providing information critical to improve diagnostic accuracy and therefore clinical management. In this regard, this study suggests that correct awareness of the prevalence of infectious diseases and the implementation of a few inexpensive diagnostic tools and a thorough evaluation of cases clinically thought to be eclampsia may lead to a significant reduction in the number of diagnostic errors [42]. The implementation of morbidity and mortality conferences may also have an important effect on maternal mortality in sub-Saharan Africa [43]. Additional studies are needed to assess whether analysis of how and when diagnostic errors occur might improve epidemiological data in maternal mortality cases and clinical diagnostic performance.

In addition to their intrinsic clinical relevance, missed diagnoses detected at autopsy may have important implications for research. The main source of information on the causes of maternal deaths in developing countries is VA, an established method of ascertaining the likely causes of death by interpretation of interviews with relatives or carers of the deceased. VAs are not based on clinical or laboratory measures and have been questioned because they are subject to a relatively high degree of misclassification error [44,45]. Our data indicate that not only VAs but also medical records may contain substantial inaccuracies regarding the main diagnoses causing or contributing to death. It is noteworthy that studies based on these methodologies tend to underreport infectious diseases [2,46]. Since diagnoses and causes of death are determined without autopsy in the vast majority of cases, especially in sub-Saharan-Africa, vital statistics, clinical registries, and even randomized trials, may capture incorrect causes of death. These inaccuracies have important policy implications, as major funding and policy decisions are derived in part from vital statistics and other registries of disease burden.

## Abbreviations

CI: confidence interval
NPV: negative predictive value
PPV: positive predictive value
VA: verbal autopsy
